# Genome variations account for different response to three mineral elements between *Medicago truncatula* ecotypes Jemalong A17 and R108

**DOI:** 10.1186/1471-2229-14-122

**Published:** 2014-05-06

**Authors:** Tian-Zuo Wang, Qiu-Ying Tian, Bao-Lan Wang, Min-Gui Zhao, Wen-Hao Zhang

**Affiliations:** 1 State Key Laboratory of Vegetation and Environmental Change, Institute of Botany, the Chinese Academy of Sciences, Beijing, P. R. China; 2 Research Network of Global Change Biology, Beijing Institutes of Life Science, the Chinese Academy of Sciences, Beijing, P. R. China

**Keywords:** Resequencing, *Medicago truncatula*, Aluminum toxicity, Aluminum- activated citrate transporter, Salt stress, *MtZpt2-1*, Iron deficiency, *Yellow Stripe-Likes*

## Abstract

**Background:**

Resequencing can be used to identify genome variations underpinning many morphological and physiological phenotypes. Legume model plant *Medicago truncatula* ecotypes Jemalong A17 (J. A17) and R108 differ in their responses to mineral toxicity of aluminum and sodium, and mineral deficiency of iron in growth medium. The difference may result from their genome variations, but no experimental evidence supports this hypothesis.

**Results:**

A total of 12,750 structure variations, 135,045 short insertions/deletions and 764,154 single nucleotide polymorphisms were identified by resequencing the genome of R108. The suppressed expression of *MtAACT* that encodes a putative aluminum-induced citrate efflux transporter by deletion of partial sequence of the second intron may account for the less aluminum-induced citrate exudation and greater accumulation of aluminum in roots of R108 than in roots of J. A17, thus rendering R108 more sensitive to aluminum toxicity. The higher expression-level of *MtZpt2-1* encoding a TFIIIA-related transcription factor in J. A17 than R108 under conditions of salt stress can be explained by the greater number of stress-responsive elements in its promoter sequence, thus conferring J. A17 more tolerant to salt stress than R108 plants by activating the expression of downstream stress-responsive genes. YSLs (Yellow Stripe-Likes) are involved in long-distance transport of iron in plants. We found that an *YSL* gene was deleted in the genome of R108 plants, thus rendering R108 less tolerance to iron deficiency than J. A17 plants.

**Conclusions:**

The deletion or change in several genes may account for the different responses of *M. truncatula* ecotypes J. A17 and R108 to mineral toxicity of aluminum and sodium as well as iron deficiency. Uncovering genome variations by resequencing is an effective method to identify different traits between species/ecotypes that are genetically related. These findings demonstrate that analyses of genome variations by resequencing can shed important light on differences in responses of *M. truncatula* ecotypes to abiotic stress in general and mineral stress in particular.

## Background

Legume is the second most important crop family in the world, and is one of primary sources for the consumption of human and animals [1,2]. Acquisition of nutrients from soil is a prerequisite for plant growth and development. Plants are frequently exposed to adverse mineral stress in soils, including aluminum toxicity in acid soil, salt stress in saline soil and iron deficiency in alkaline soil. Plants have evolved numerous mechanims to adapt to these stressed environments [3-5]. Understanding of the molecular mechanims by which plants respond and adapt to the mineral toxicity and deficiency is a major challenge in modern plant biology.

As a model legume species, *Medicago truncatula* Gaertn has been widely used to study functional genomics because of its small diploid genome, self-fertility, short generation cycle and easy transformation [6]. There are a number of ecotypes of *M. truncatula* with large genetic variations [7]. Of the ecotypes, *M. truncatula* ecotype Jemalong A17 (J. A17) has been used for the whole-genome sequencing and physiological studies [8-10], while ecotype R108 is often used for gene transformation because of its superior *in vitro* regeneration [11]. *M. truncatula* ecotype R108 differs from its counterpart J. A17 in traits associated with development, and biotic/abiotic responses. For example, treatments of J. A17 with methyl jasmonate and ethylene induce resistance to fungal pathogen *Macrophomina phaseolina*, while these treatments fail to induce resistance in R108 to the fungal pathogen [12]. In addition, rhizobial-induced expression of chitinase gene between the two ecotypes is also different [13]. The two ecotypes exhibit different tolerance to salt stress, such that ecotype J. A17 is more tolerant to salt stress than R108. Further studies reveal that a TFIIIA-related transcription factor gene, *MtZpt2-1* shows different expression in the two ecotypes, and that overexpression of *MtZpt2-1* in roots confers enhanced tolerance to salt stress [14,15]. Our previous work revealed that the two ecotypes also differed in their tolerance to deficiency in mineral nutrients. For example, ecotype J. R108 was more sensitive to iron deficiency than ecotype J. A17 [16]. Despite the morphological and physiological differences between the two ecotypes, few studies have investigated the molecular mechanisms underlying the differences due to lack of information on the genome of R108.

DNA sequences contain all the genetic information, and genome variations such as structure variations (SVs), short insertions/deletions (indels) and single nucleotide polymorphisms (SNP) can explain many variations in morphological, physiological, and ecological traits [17-21]. Resequencing technology provides a powerful tool to study these variations among species/ecotypes that are closely related genetically. For instance, *Thellungiella salsuginea* exhibits exceptionally high resistance to cold, drought, and oxidative stresses as well as salinity [22-24]. The number of members in gene families with known functions associated with responses to abiotic stresses in *T. salsuginea* is greater than in *Arabidopsis thaliana*, including those gene families of *RAV*, *NF-X1*, *GRAS*, *HSF*, *HKT*, *CIPK* and *CDPK*[25]. Furthermore, it has been reported that maize inbred-line Mo17 exhibits eminent heterosis due to its deletion of eighteen genes [19]. The two widely used *M. truncatula* ecotypes Jemalong A17 (J. A17) and R108 have been reported to differ in their tolerance to salt stress [14,15] and iron deficiency [16]. To test whether the genome variations between J. A17 and R108 may account for the differences in their responses to mineral toxicity of aluminum and sodium and mineral deficiency of iron in growth medium, genome variations of *M. truncatula* ecotype R108 were analyzed by mapping the reads obtained from resequencing of R108 to the reference genome of ecotype J. A17.

## Results

### Response of J. A17 and R108 to Al^3+^ and Na^+^ toxicity, and Fe deficiency

**Figure 1:**
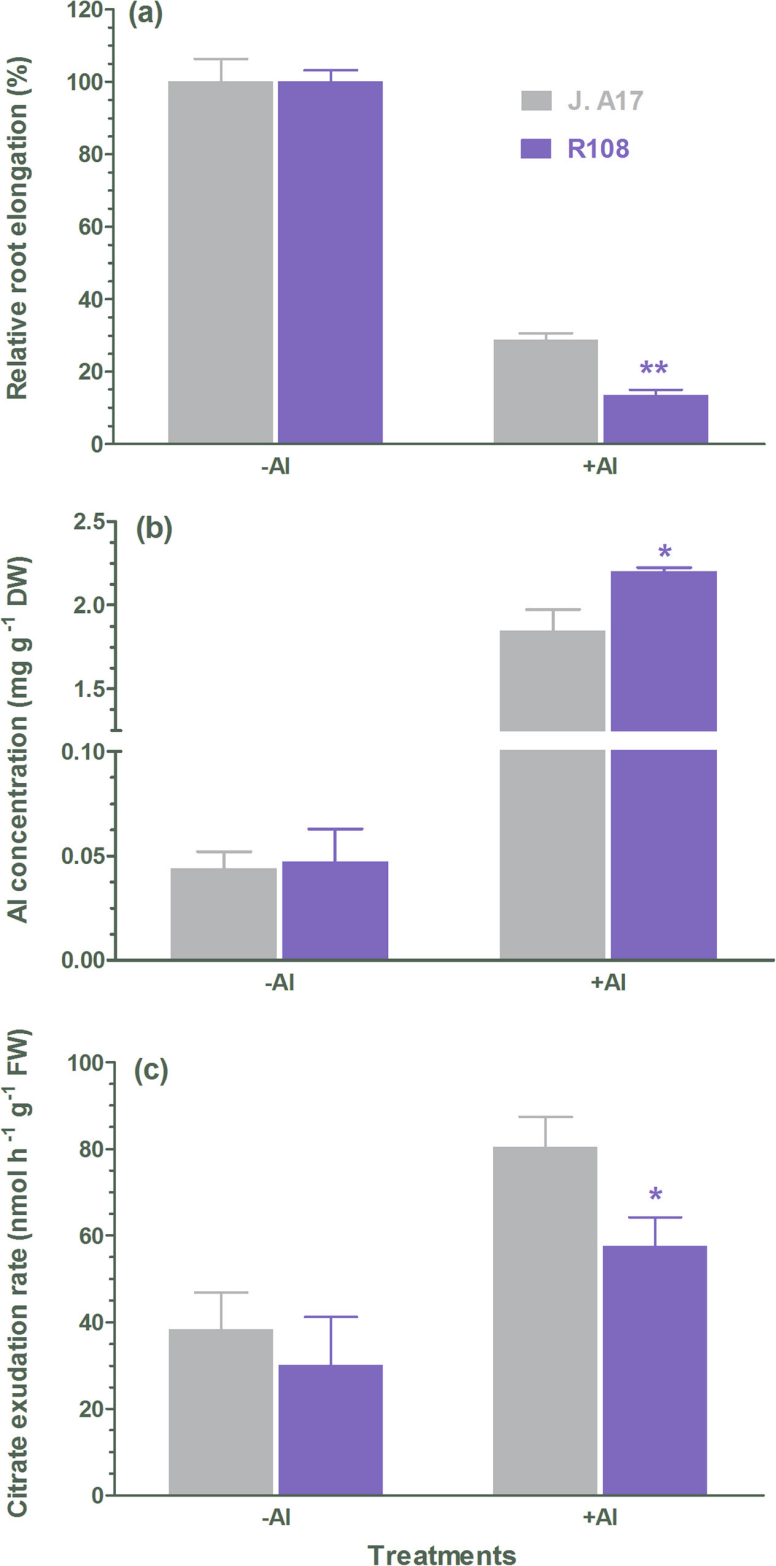
**Effect of Al**^**3+ **^**on root elongation, citrate exudation and Al content in roots of J. A17 and R108 plants.** The relative root elongation was determined by exposing 3-d-old seedlings of J. 17 and R108 to 5 μM AlCl_3_ (pH 4.5) for 2 days **(a)**. Data are mean ± s.e. with *n* = 10. Al contents in roots of J. A17 and R108 plants before and after exposure to 5 μM AlCl_3_ (pH 4.5) for 2 days **(b)**. Data are mean ± s.e. with *n* = 4. Citrate exudation rate from roots of J. A17 and R108 plants treated with 5 μM AlCl_3_ (pH 4.5) for 24 h **(c)**. Data are mean ± s.e. with *n* = 5. * and ** indicate significant difference between genotypes within a given growth condition (−Al or + Al) at *P* ≤ 0.05 and *P* ≤ 0.01, respectively.

**Figure 2:**
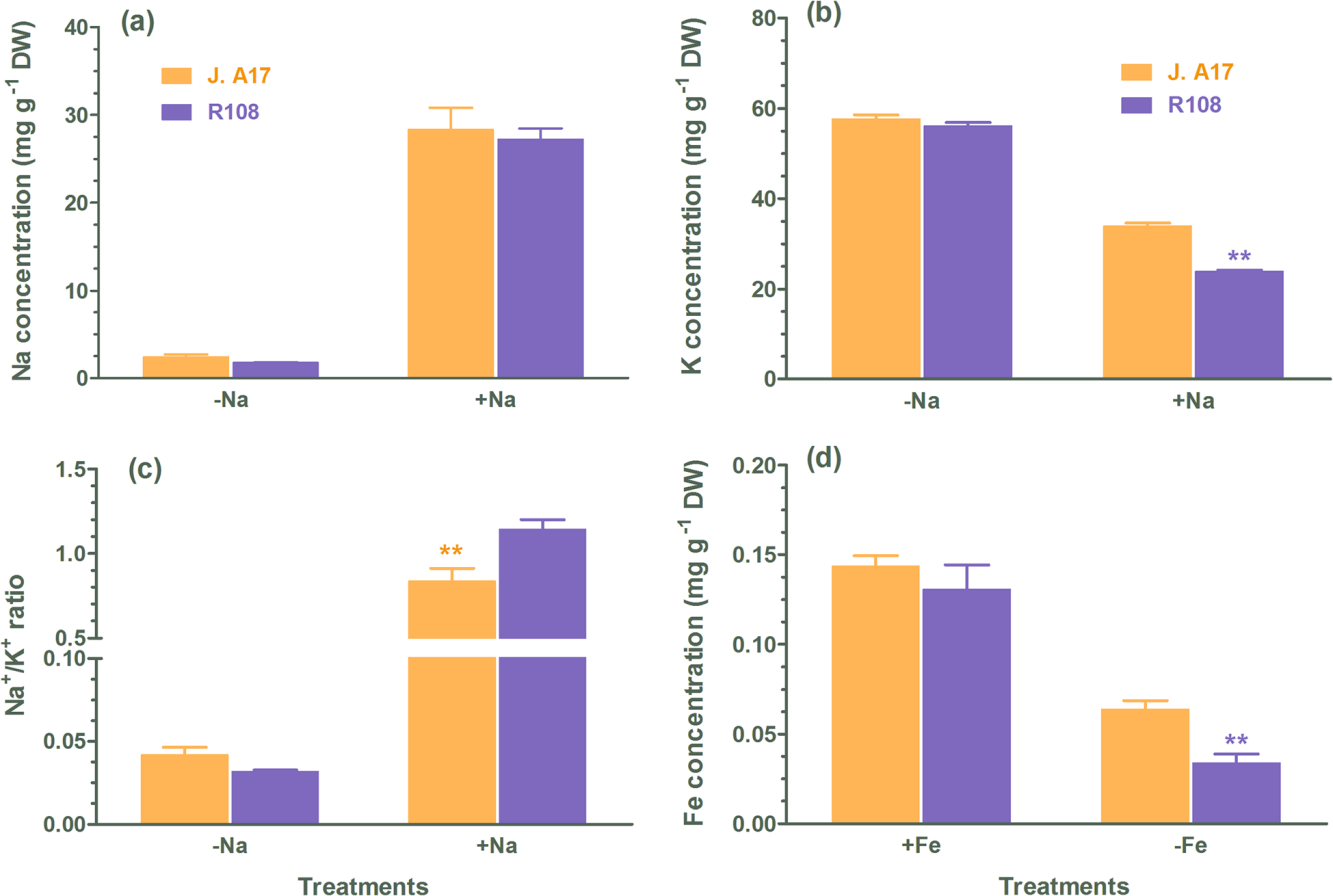
**Effects of salt stress and iron deficiency on Na**^**+ **^**and K**^**+ **^**concentrations, Na**^**+**^**/K**^**+ **^**ratio, and Fe concentrations in shoots of J. A17 and R108 plants.** Concentrations of Na and K and Na^+^/K^+^ ratio in shoots treated with and without 100 mM NaCl for 5 days were shown in panel **(a)**, **(b)** and **(c)**, respectively. Data are mean ± s.e. with *n* = 4. Fe concentration in shoots of 5-d-old seedlings of J. A17 and R108 plants exposed to control, Fe-sufficient medium (100 μM Fe-EDTA, +Fe) and Fe-deficient medium (1 μM Fe-EDTA) for 5 days **(d)**. * and ** indicate significant difference between genotypes within a given growth condition at *P* ≤ 0.05 and *P* ≤ 0.01, respectively.

To examine the effect of Al^3+^ on root elongation of the two ecotypes, the relative root elongation was determined. As shown in Figure 1a, root elongation was inhibited upon exposure of the two ecotypes to solution containing Al^3+^, and the Al^3+^-induced inhibition of root elongation in R108 was greater than in J. A17. Moreover, Al contents in R108 roots were higher than in J. A17 roots (Figure 1b), implying that an exclusion mechanism may operate in ecotype J. A17 plants. Exudation of organic anions including malate and citrate to complex toxic Al^3+^ in the rhizosphere is an important mechanism to tolerate Al [3,26,27]. Therefore, we monitored exudate of malate and citrate from roots of the two ecotypes in response to Al^3+^ treatment. There was an increase in citrate exudation from roots of J. A17 and R108 plants by exposure to Al^3+^, and the Al^3+^-induced citrate exudation from roots of J. A17 was greater than that of R108 plants (Figure 1c). In contrast to citrate, no significant increases in malate exudation from roots of the two ecotypes by exposure to Al^3+^ were detected (data not shown). These results suggest that higher exudation of citrate may underpin the greater tolerance of J. A17 to Al than R108 plants. de Lorenzo *et al*. found that R108 is more sensitive to salt stress than J. A17 plants as evidenced by less suppression of root growth in J. A17 plants than in R108 plants [14]. In addition to root growth, Na^+^/K^+^ ratio is an important indicator for tolerance of plants to salt stress. Excessive accumulation of toxic Na^+^ in plant cells, particularly in the cytosol, disrupts K^+^ homeostasis, leading to dysfunction of plant cells, thus plants displaying high tolerance to salt stress often minimize Na^+^ uptake and/or maximize K^+^ acquisition to maintain a low Na^+^/K^+^ ratio [28]. Therefore, we compared the effect of salt stress on Na^+^ and K^+^ concentrations in the two ecotypes. No differences in both Na^+^ and K^+^ concentrations in shoots of the two ecotypes were found when they were grown in the control medium (Figure 2a and b). When they were exposed to solution containing NaCl, an enhanced accumulation of Na^+^ in both ecotypes was observed (Figure 2a). However, exposure to salt stress led to reductions in K^+^ concentrations in shoots of both ecotypes, and the salt stress-induced reduction in K^+^ concentration was greater in R108 than in J. A17 plants (Figure 2b). This led to an increase in Na^+^/K^+^ ratio in both ecotypes, and the increase was significantly less in J. A17 plants than in R108 plants (Figure 2c).

Our previous work showed that the ecotype J. A17 was more tolerant to Fe deficiency than R108 by efficiently mobilizing Fe in the rhizosphere and transporting of Fe from roots to shoots in J. A17 plants [16]. A similar result showing that ecotype J. A17 had higher foliar Fe contents than R108 when grown in Fe-deficient medium was observed in the present study (Figure 2d). These results show that the two ecotypes differ in their tolerance to toxicities of Al and Na as well as Fe deficiency by differently regulating citrate exudation, Na uptake and Fe transport, respectively.

### Resequencing of R108

**Figure 3:**
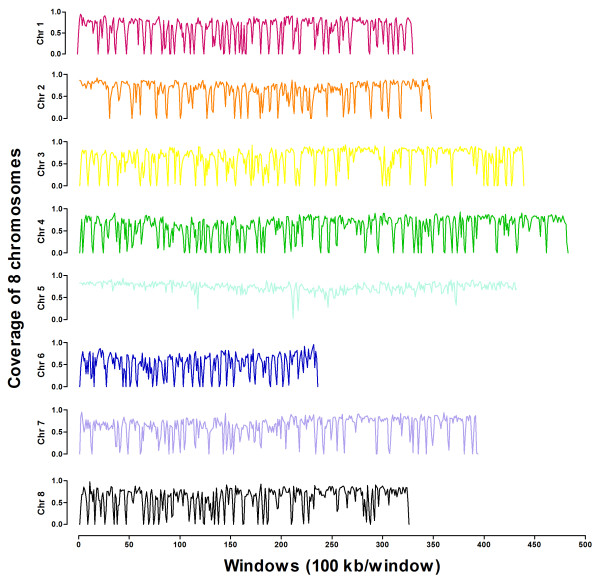
**The sequencing coverage of 8 chromosomes in the genome of R108 against to the reference of J. A17 genome.** One hundred kb was defined as one window.

Paired-end sequencing method was employed to resequence the genome of *M. truncatula* ecotype R108, and about 4.64 Gb original sequencing data were generated. High-quality reads of 4.28 Gb were obtained after initially processing. The genome of R108 is 17% smaller than that of J. A17 [29]. This led to a sequencing mean coverage and depth of approx. 72% and 11-fold over the whole genome, respectively (Figure 3 and Additional file 1: Figure S1). The coverage of chromosome 5 was the greatest among the chromosomes. In addition, we found a similar coverage of chromosome 5 in a tetraploid *Medicago falcata* (unpublished results), suggesting that Chr 5 may be the most conserved in the genus of Medicago.

Structure variations (SVs), short insertions/deletions (indels) and single nucleotide polymorphisms (SNPs) were identified by aligning the high-quality sequences against the reference genome of J. A17. We obtained a total of 12,750 SVs, 135,045 indels and 764,154 SNPs in the genome of R108 (Table 1).

**Table 1:** The number of SVs, indels and SNPs in the R108 genome

**Genome variations**		**Numbers**
SV	Insertion	1,239
	Deletion	10,964
	Others	547
Indel	Insertion	67,087
	Deletion	67,958
SNP	Homozygosity	660,168
	Heterozygosity	103,986

The data were obtained by mapping the reads of R108 obtained from resequencing to the reference genome of ecotype J. A17. SVs were separated into insertions, deletions and others. Indels were separated into insertions and deletions. SNPs were separated into homozygosity and heterozygosity.

Structure variations are important types of differences among individuals of the same species, and can cause large alterations to the genome, resulting in the differences in phenotypes. We identified 10,964 deletions, 1,239 insertions and 547 other SVs such as duplication, inversion and transposition by resequencing (Table 1). The quantity of deletions was more abundant than other SVs. This result is consistent with the forecast as the genome of R108 has been reported to be smaller than that of J. A17 [29]. We also identified 135,045 indels ranging from 1–5 bp in length. Among these short indels, the number of insertions and deletions was almost equal (Table 1). Insertion of one bp and deletion of one bp were the mostly observed insertions and deletions, respectively, accounting for more than half of the total number of insertions and deletions (Additional file 1: Figure S2). Generally, genome variations were mainly accounted for by SNPs. Eighty-six percent of SNPs were homozygous over the whole genome (Table 1). For the SNPs within coding sequences, there were 70,695 nonsynonymous and 57,124 synonymous SNPs, respectively. This led to a ratio of nonsynonymous to synonymous nucleotide (Nonsyn/Syn) of 1.24. A similar ratio has been reported in soybean and rice, while the ratio in Arabidopsis (0.83) is smaller than our finding in the present study [17,18,30].

### Variations of mineral element-related genes

The resequencing data obtained from *M. truncatula* ecotype R108 revealed that some genes involved in acquisition of mineral elements were deleted in R108 plants compared to ecotype J. A17 (Table 2).

**Table 2:** **Deleted genes related to mineral stress in the genome of *M. truncatula *** **ecotype R108 plants**

**Genes**	**Positions in reference**	**Annotations**	**Positions of deletions**
*Medtr8g036660*	MtChr8: 8363576-8369153 (−)	Putative aluminum activated citrate transporter	MtChr8: 8367361-8368131
*Medtr1g007540*	MtChr1: 996073–999271 (−)	Metal-nicotianamine transporter YSL3	MtChr1:980798-1010225

The data were obtained by mapping the reads of R108 obtained from resequencing to the reference genome of ecotype J. A17.

**Figure 4:**
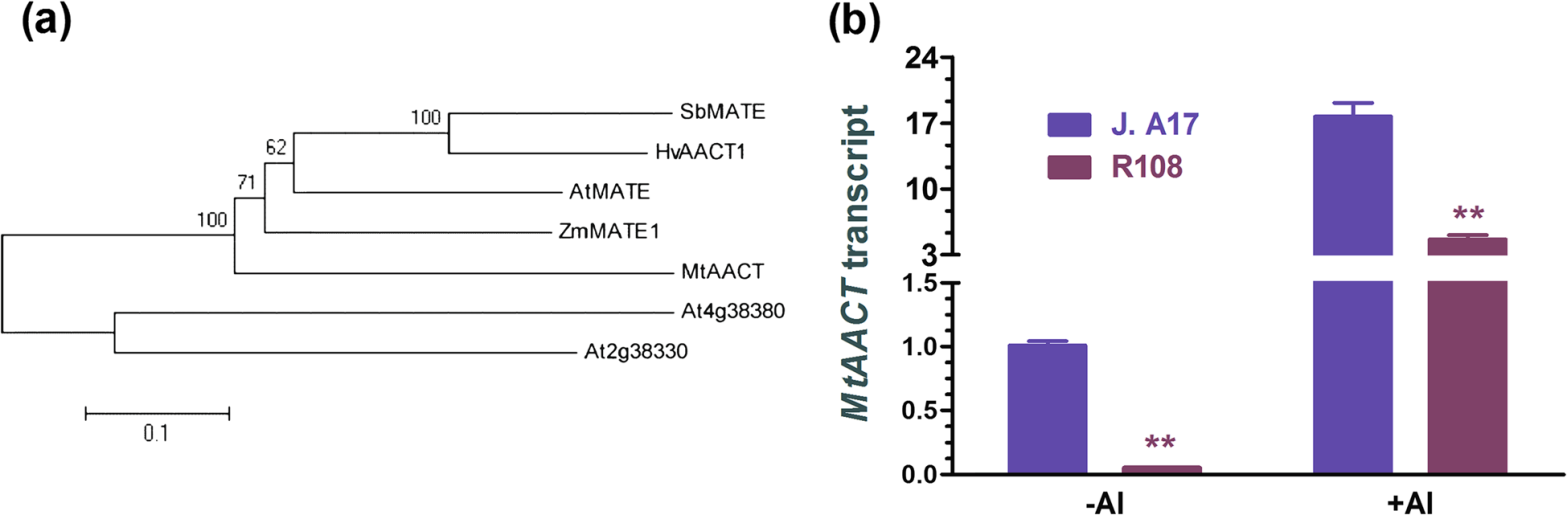
**Similarity of MtAACT protein to other known AACT proteins and effect of Al**^**3+ **^**on expression of MtAACT in J. A17 and R108 plants.** Phylogenetic tree of known and putative Al-activated citrate transporters was constructed by MEGA 5 in panel **(a)**. The accession numbers of SbMATE, HvAACT1, AtMATE, ZmMATE1, MtMATE, At4g38380 and At2g38330 in GenBank are ABS89149.1, BAF75822.1, NP_974000.1, ACM47311.1, XP_003627698.1, NP_195551.5 and NP_181367.2, respectively. The expression of *MtAACT* in roots of J. A17 and R108 plants under the conditions of with or without 5 μM A1Cl_3_ (pH 4.5) in medium for 1 days **(b)**. Data are mean ± s.e. with three biological replicates. * and ** indicate significant difference between genotypes within a given growth condition (−Al or + Al) at *P* ≤ 0.05 and *P* ≤ 0.01, respectively.

Aluminum-induced exudation of citrate from roots that is mediated by membrane transporters can detoxify toxic Al^3+^ in the rhizosphere by forming non-toxic Al-citrate complex [3,26,27]. Several genes encoding the transporters of Al-induced citrate exudation belonging to MATE family have been identified [31-34]. Our resequencing data show that 771 bp in the second intron of a gene encoding a putative aluminum-activated citrate transporter (MtAACT) was deleted (Table 2, Additional file 1: Figure S3). The sequence of MtAACT was similar with the known Al-induced citrate transporters (Figure 4a). Expression-level of *MtAACT* in J. A17 was higher than in R108 in the absence of Al^3+^, and it was up-regulated in both ecotypes by exposure to AlCl_3_ with the Al-induced expression of *MtAACT* in R108 being lower than in J. A17 plants (Figure 4b).

**Figure 5:**
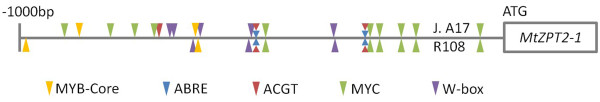
**Analysis of *MtZpt2-1 *promoter sequence of J. A17 and R108.** The arrows above line represent *cis*-elements of J. A17, and that of below the line indicate elements of R108.

Previous studies have shown that R108 is more sensitive to salt stress than J. A17, and real-time qPCR showed that expression of *MtZpt2-1* is greater in J. A17 than in R108 plants [14]. Overexpression of *MtZpt2-1* in roots of the salt-sensitive ecotype of *M. truncatula* confers enhanced tolerance to salt stress, suggesting that differential expression of *MtZpt2-1* is responsible for the difference in adaptation to salt stress. Resequencing allowed us to analyze the promoter sequence of *MtZpt2-1* in R108 plants. Stress-responsive-related *cis*-elements were identified by PLACE database between J. A17 and R108 (Figure 5). The number of MYC and W-box elements was greater in J. A17 than in R108, which may underpin the higher expression levels of *MtZpt2-1* in J. A17 than in R108 plants under conditions of salt stress.

**Figure 6:**
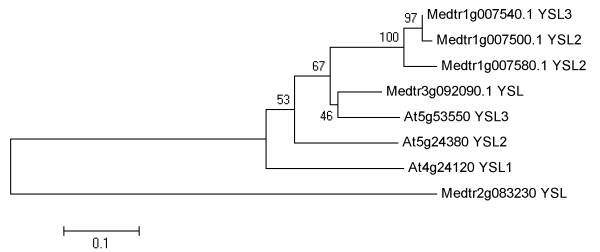
**Sequence analysis of YSL protein family.** Phylogenetic tree of these proteins was constructed by MEGA 5. The corresponding IDs were shown in the figure.

YSLs (Yellow Stripe-Likes) are involved in long-distance transport of Fe in plants [35,36]. There are five YSLs in the genome of *M. truncatula* according to Mt3.5 assembly of the reference genome. Our resequencing results show that an *YSL* gene (*Medtr1g007540*) was deleted in the genome of R108 (Table 2). The protein encoded by *Medtr1g007540* is highly similar to Arabidopsis AtYSL3 (At5g53550) (Figure 6). The deletion of the *YSL* gene in the genome of R108 may account for the less accumulation of iron in the shoots of R108 (Figure 2d).

## Discussion

### Identification of genome variations using resequencing

Analyses of gene expression by methods such as transcriptome, microarray and DGE have been used to decipher the differential responses among species and cultivars/ecotypes with close genetic background to abiotic stresses [14,37-40]. However, these methods are less effective when several mechanisms underlie the different responses to abiotic stresses. Moreover these methods cannot be used to analyze *cis*-acting regulatory elements. In contrast, resequencing technology can identify genome variations which are responsible for morphological and physiological differences [41]. In addition, *cis*-acting regulatory sequences obtained from the resequencing can be used to pinpoint the differential expression in response to abiotic stresses. Estimation of phylogenetic relationships among Medicago species by genome resequencing has been reported [42]. However, genome resequencing has not been used to investigate responses of Medicago species to abiotic stresses in general and mineral stresses in particular so far. In the present study, we utilized this technology to decipher the mechanisms underlying the different responses of two *M. truncatula* ecotypes to aluminum toxicity, salt stress and iron deficiency.

### Tolerance of *M. truncatula* to Al is achieved by citrate exudation

Aluminum is the most abundant metal in the earth’s crust. Phytotoxic Al^3+^ is solubilized when soil becomes acidified. Inhibition of root elongation is one of the earliest and most distinct symptoms exhibited by plants suffering from Al toxicity [43]. Plants have evolved numerous mechanisms to adapt to Al toxicity. Exudation of organic anions from root apices to chelate toxic Al^3+^ in the rhizosphere is an effective way to detoxify Al toxicity, thus conferring tolerance to Al toxicity [3,26,27]. Several Al-activated citrate transporters have been shown to be involved in regulation of Al tolerance. For instance, *SbMATE* in sorghum (*Sorghum bicolor*) and *HvAACT1* in barley (*Hordeum vulgare*) that belong to the multidrug and toxic compound exudation (MATE) family have been identified to mediate Al-activated citrate exudation. Heterologous expression of *SbMATE* in Arabidopsis and *HvAACT1* in tobacco leads to enhanced citrate efflux, thus conferring tolerance to Al toxicity [31,32]. The homologs in Arabidopsis and maize have subsequently been cloned [33,34].

In the present study, we found that root elongation of R108 was more inhibited by Al than that of J. A17 plants (Figure 1a), suggesting that R108 is more sensitive to Al than J. A17. We uncovered deletion of partial sequence in the second intron of a gene encoding a putative Al-activated citrate transporter (MtAACT) in R108 plants by resequencing (Table 2). The amino acid sequence of this transporter is similar with the known Al-activated citrate transporters in other plant species (Figure 4a). In addition, expression of this gene was up-regulated by Al in both ecotypes with the magnitude of Al-induced expression of *MtAACT* in R108 less than in J. A17 plants (Figure 4b), suggesting that expression of *MtAACT* is sensitive to Al^3+^. The suppressed expression of *MtAACT* in R108 relative to that in J. A17 plants is likely to be accounted for by the deletion of the partial sequence of the second intron. The intron deleted in R108 plants may activate gene expression by enhancers within introns and/or regulating chromatin remodeling. Several introns in plants are reported to increase the expression of genes. For example, the second intron from Arabidopsis *agamous* gene can function in both orientations to drive expression of reporter gene from a minimal promoter [44]. The first intron of Arabidopsis gene encoding elongation factor eEF-1β has the similar function to enhance gene expression [45]. The lower abundance of *MtAACT* transcripts in R108 than J. A17 when exposed to solution containing toxic Al^3+^ may explain the less citrate released from roots of R108 than J. A17 plants in response to Al treatment (Figure 1c). The reduced citrate exudation from roots of R108 plants due to reduced expression of *MtAACT* would render R108 plants less effective to complex toxic Al^3+^ in the rhizosphere, thus making it less tolerant to Al than J. A17 plants. The greater accumulation of Al in roots of R108 than in those of J. A17 is in line with this argument (Figure 1b).

### Promoter analysis of *MtZpt2-1*

The ecotype J. A17 plants have been shown to be more tolerant to salt stress than R108 plants [14,15]. A gene encoding a TFIIIA-related transcription factor, *MtZpt2-1* has been identified by its greater up-regulation in J. A17 than R108 plants under salt stress [14]. MtZpt2-1 can active the expression of many stress-responsive genes [46]. Several stress-related *cis*-elements were found by analyzing the promoter sequences of *MtZpt2-1* in both ecotypes (Figure 5). These stress-related *cis*-elements in *MtZpt2-1* can allow this gene to be up-regulated in response to abiotic stresses, thus participating in the regulation of tolerance to abiotic stresses. Two MYB-core elements and ABA-responsive elements (ABRE) have been shown to be involved in responses to osmotic stress and ABA, respectively [47,48]. However, the two ecotypes differed in their promoter sequences of *MtZpt2-1,* such that *MtZpt2-1* of J. A17 plants had one more ACGT element, four more MYC elements and two more W-box elements than R108 plants. There are reports showing the involvements of these *cis*-elements in stress response [49-51]. The greater number of *cis*-elements of *MtZpt2-1* in J. A17 plants may explain higher expression of *MtZpt2-1* in J. A17 plants than in R108 plants under conditions of salt stress, thus conferring their tolerance to salt stress.

### Function of YSL in iron transport

YS1 (Yellow Stripe 1) has been identified to be involved in uptake of iron from soil by roots in maize [52,53]. Based on their sequence similarity to the maize YS1, eight YSLs (Yellow Stripe-Likes) were identified in Arabidopsis. *AtYSL1*, *AtYSL2* and *AtYSL3* are expressed most strongly in the vascular parenchyma cells [36,54]. The *ysl1ysl3* double mutant displays strong interveinal chlorosis, and has reduced foliar iron content [36]. These findings suggest that YSLs act as key mediators in unloading iron to mesophyll cell after iron is transported from roots through xylem in plants [55].

Five YSLs were identified in the genome of Medicago according to Mt3.5. However, in the genome of R108, an *YSL* gene (*Medtr1g007540*) was deleted (Table 2). The protein encoded by the *YSL* gene had high similarity with AtYSL3 of Arabidopsis (Figure 6). We hypothesize that this protein may be involved in unloading of iron from the vascular tissues to mesophyll cells. The deletion of this gene in R108 plants would impair iron unloading to mesophyll cells, thus leading to the reduced iron contents in shoots of R108 plants when grown in iron-deficient medium (Figure 2d).

## Conclusions

The two *M. truncatula* ecotypes Jemalong A17 and R108 differed in their sensitivity to aluminum toxicity, salt stress and iron deficiency. Resequencing of *M. truncatula* ecotype R108 uncovered a total of 12,750 SVs, 135,045 indels and 764,154 SNPs by comparing with the reference genome of J. A17. We found that the partial sequence of the second intron of *MtAACT* that encodes a putative Al-activated citrate transporter was deleted. This partial deletion may lead to the lower expression level of *MtACC*T in R108 plants than that in J. A17 plants in the absence and presence of toxic Al in the growth medium. The reduced expression of *MtAACT* in R108 plants in turn may render less exudation of citrate form roots to detoxify Al in the rhizosphere, thus making R108 plants less tolerance to Al than J. A17 plants. In addition, we demonstrated that promoter sequence in *MtZpt2-1* of J. A17 plants contained more response-elements than that of R108 plants. Given the regulatory roles of *MtZpt2-1* in response to salt stress, these results may account for the greater tolerance of J. A17 plants to salt stress than R108 plants. Finally, our results revealed that deletion of an *YSL* gene encoding an iron transporter in the genome of R108 plants is likely to impair long-distance transport of iron in R108 plants. This result may explain the greater sensitivity of R108 plants to iron deficiency than J. A17 plants. Taken together, these findings demonstrate that analyses of genome variations by sequencing can shed important light on differences in responses of *M. truncatula* ecotypes to abiotic stress in general and mineral stress in particular.

## Methods

### Plant materials and treatments

Two *Medicago truncatula* ecotypes Jemalong A17 and R108 were used in this study. Seeds of both ecotypes were treated with concentrated sulfuric acid for 8 min, and then thoroughly rinsed with water. After chilled at 4°C for 2 d, seeds were sown on 0.8% agar to germinate at 25°C until the radicals were approximately 2 cm. The seeds were planted in the same plastic buckets (6 seedlings for both ecotypes per bucket) filled with 2.5 L aerated nutrient solution. The composition of full-strength nutrient solution is: 2.5 mM KNO_3_, 0.5 mM KH_2_PO_4_, 0.25 mM CaCl_2_, 1 mM MgSO_4,_ 100 μM Fe-Na-EDTA, 30 μM H_3_BO_3_, 5 μM MnSO_4_, 1 μM ZnSO_4_, 1 μM CuSO_4_ and 0.7 μM Na_2_MoO_4_ with pH of 6.0.

For measurements of the effect of AlCl_3_ on root elongation, 3-d-old seedlings were transferred into solutions containing 0.5 mM CaCl_2_ with and without 5 μM AlCl_3_ (pH 4.5) for 2 days. Length of primary root was measured after treatment with AlCl_3_, and relative root elongation was calculated. To determine the effect of AlCl_3_ on exudation of citrate from roots, three-week-old seedlings were transferred into solutions containing 0.2 mM CaCl_2_ with and without 5 μM AlCl_3_ (pH 4.5) for 1 days. The exudation from the treated roots was collected at room temperature without light for 2 hours, and then citrate concentration in the exudation solution was determined by reversed-phase high performance liquid chromatography (HPLC) as described previously [56]. For measurements of Al content in roots, seedlings of the two ecotypes were treated with 5 μM AlCl_3_ (pH 4.5) for 24 h, and roots were collected for measurement of Al.

Three-week-old seedlings were transferred into solutions containing 100 mM NaCl or 1 μM Fe-Na-EDTA for 5 days. Shoots were collected to measure the content of Na^+^, K^+^ and Fe.

### Measurement of mineral elements

Plant materials treated with and without mineral stress (Al and Na toxicity and Fe deficiency) were harvested and dried at 80°C to constant weight. As much as 50 mg of dry plant material was weighed and placed in a digestion tube, and then samples were digested with 6 mL of nitric acid and 2 mL of hydrogen peroxide using microwave system (MARS, CEM). The digest were diluted to 50 mL. After filtering, the concentrations of Al, Na, K and Fe were measured by ICP-AES (Thermo).

### DNA isolation and resequencing

DNA isolation was carried out using a CTAB (cetyl trimethylammonium bromide) protocol. After quality assay, genomic DNA was fragmented randomly. After electrophoresis, DNA fragments of about 500 bp were gel purified. Adapter ligation and DNA cluster preparation were performed and subjected to 2 × 90 bp paired-end sequencing on an Illumina Hiseq2000 sequencer. The raw data have been submitted to NCBI Sequence Read Archive (http://www.ncbi.nlm.nih.gov/sra) and the accession number is SRP029924.

### Bioinformatics analysis

Firstly, adapter contamination in the raw data was removed. To ensure quality, each base in a read was assigned a quality score (Q) by a phred-like algorithm [57,58]. The reads which contained more than 50% low quality bases (Q ≤ 5) were removed. Using SOAP2 [59], all reads were aligned with the *M. truncatula* reference genome (Mt 3.5 assembly) [10]. If the original read could not be aligned onto the reference sequence, the first nucleotides at 5’ end and two nucleotides at 3’ end were deleted, and then aligned onto the reference again. If the sequence failed to alignment, two more nucleotides at 3’ end were deleted. The procedure was repeated until alignment was achieved or the read was less than 32 bp. The average sequencing depth and coverage was calculated using the results of alignment.

Structure variations, short indels and SNPs were identified by aligning the reads of R108 obtained from resequencing to the reference genome of ecotype J. A17. In our experiment, the distance of both relevant paired-end reads should be about 500 bp. However, if the distance and orientation were different from expectation after both relevant paired-end reads were aligned with the reference genome, the region might have variation structures. The types of structure variations that can be detected include deletion, insertion, duplication, inversion and transposition. SOAPsv was used to identify structure variation, and at least three paired-end reads were needed to confirm a variation structure in the present study. The alignment gaps in mapped reads were identified as candidate indels using SOAPindel. The maximum gap length was 5 bp, and at least three pairs of reads to define an indel. On the basis of alignment, polymorphic loci against the reference sequence were identified according to the following criteria: Q ≥ 20, 3 ≤ Depth ≤ 100 and at least 5 bp away from each other. SOAPsnp was used in this assay.

Plant *cis*-acting regulatory elements were searched by the PLACE database [60].

### RNA isolation and real-time quantitative PCR

Total RNA was isolated using RNAiso Plus reagent (TaKaRa) and treated with RNase-free DNase I (Promega). The total RNA was reverse-transcribed into first-strand cDNA with PrimeScript® RT reagent Kit (TaKaRa).

Real-time quantitative PCR (RT-qPCR) was performed using ABI Stepone Plus instrument. Gene-specific primers of *MtAACT* (accession No. XM_003627650.1) were 5'-GAC ATA GAG AAA GGG ACA-3' and 5'-AGG ATA GTA AAT GGG GTT-3'. *MtActin* (accession No. BT141409) and *MtGADPH* (accession No. XM_003608827.1) were used as internal control with primers: (5'-ACG AGC GTT TCA GAT G-3' and 5'-ACC TCC GAT CCA GAC A-3') and (5'- AAG GAG GAG TCT GAG GGC-3' and 5'-AAC GGC TGC TAG GCT AAT-3'). Each reaction contained 5.0 μL of SYBR Green Master Mix reagent (TOYOBO), 0.4 μL cDNA samples, and 0.6 μL of 10 μM gene-specific primers in a final volume of 10 μL. The thermal cycle used was 95°C for 2 min, 40 cycles of 95°C for 30 s, 55°C for 30 s, and 72°C for 30 s. The relative expression level was calculated by the comparative C_T_ method.

## Competing interests

The authors declare that they have no competing interests.

## Authors’ contributions

TZW WHZ designed the experiments; TZW conducted the experiments; TZW QYT BLW MGZ WHZ analyzed the data; TZW WHZ wrote the paper. All authors read and approved the final manuscript.

## Supplementary Material

Additional file 1: Figure S1 The R108 sequencing depth of 8 chromosomes against to the reference J. A17. One hundred kb was defined as one window. The points with depth more than 15 were hided to make the figure clearer. **Figure S2.** The number of indels varying from 1 to 5 bp in the genome of R108. The number of insertions and deletions varying from 1 to 5 bp was shown in panel (a) and (b), respectively. The “I” and “D” mean insertion and deletion, respectively. **Figure S3.** The structure of the *MtAACT* genomic region. The exons and introns are drawn as rectangles and lines, respectively. The region with red crosses is deleted in the genome of R108. Click here for file

## References

[B1] Graham PH Vance CP Legumes: Importance and constraints to greater use Plant Physiol 2003 131 3 872 877 10.1104/pp.017004 12644639 PMC1540286

[B2] Cook DR *Medicago truncatula* - a model in the making! Commentary Curr Opin Plant Biol 1999 2 4 301 304 10.1016/S1369-5266(99)80053-3 10459004

[B3] Ma JF Ryan PR Delhaize E Aluminium tolerance in plants and the complexing role of organic acids Trends Plant Sci 2001 6 6 273 278 10.1016/S1360-1385(01)01961-6 11378470

[B4] Thimm O Essigmann B Kloska S Altmann T Buckhout TJ Response of arabidopsis to iron deficiency stress as revealed by microarray analysis Plant Physiol 2001 127 3 1030 1043 10.1104/pp.010191 11706184 PMC129273

[B5] Munns R Tester M Mechanisms of salinity tolerance Annu Rev Plant Biol 2008 59 651 681 10.1146/annurev.arplant.59.032607.092911 18444910

[B6] Branca A Paape TD Zhou P Briskine R Farmer AD Mudge J Bharti AK Woodward JE May GD Gentzbittel L Ben C Denny R Sadowsky MJ Ronfort J Bataillon T Young ND Tiffin P Whole-genome nucleotide diversity, recombination, and linkage disequilibrium in the model legume *Medicago truncatula* Proc Natl Acad Sci U S A 2011 108 42 E864 E870 10.1073/pnas.1104032108 21949378 PMC3198318

[B7] Ellwood SR D’Souza NK Kamphuis LG Burgess TI Nair RM Oliver RP SSR analysis of the *Medicago truncatula* SARDI core collection reveals substantial diversity and unusual genotype dispersal throughout the Mediterranean basin Theor Appl Genet 2006 112 5 977 983 10.1007/s00122-005-0202-1 16402186

[B8] Rodriguez-Celma J Lin WD Fu GM Abadia J Lopez-Millan AF Schmidt W Mutually exclusive alterations in secondary metabolism are critical for the uptake of insoluble iron compounds by Arabidopsis and *Medicago truncatula* Plant Physiol 2013 162 3 1473 1485 10.1104/pp.113.220426 23735511 PMC3707556

[B9] Wang T Chen L Zhao M Tian Q Zhang WH Identification of drought-responsive microRNAs in *Medicago truncatula* by genome-wide high-throughput sequencing BMC Genomics 2011 12 367 10.1186/1471-2164-12-367 21762498 PMC3160423

[B10] Young ND Debelle F Oldroyd GED Geurts R Cannon SB Udvardi MK Benedito VA Mayer KFX Gouzy J Schoof H Van de Peer Y Proost S Cook DR Meyers BC Spannagl M Cheung F De Mita S Krishnakumar V Gundlach H Zhou SG Mudge J Bharti AK Murray JD Naoumkina MA Rosen B Silverstein KAT Tang HB Rombauts S Zhao PX Zhou P The Medicago genome provides insight into the evolution of rhizobial symbioses Nature 2011 480 7378 520 524 22089132 10.1038/nature10625 PMC3272368

[B11] Hoffmann B Trinh TH Leung J Kondorosi A Kondorosi E A new *Medicago truncatula* line with superior in vitro regeneration, transformation, and symbiotic properties isolated through cell culture selection Mol Plant Microbe In 1997 10 3 307 315 10.1094/MPMI.1997.10.3.307

[B12] Gaige AR Doerksen T Shuai B *Medicago truncatula* ecotypes A17 and R108 show variations in jasmonic acid/ethylene induced resistance to *Macrophomina phaseolina* Can J Plant Pathol 2012 34 1 98 103 10.1080/07060661.2012.662176

[B13] Salzer P Feddermann N Wiemken A Boller T Staehelin C *Sinorhizobium meliloti*-induced chitinase gene expression in *Medicago truncatula* ecotype R108-1: a comparison between symbiosis-specific class V and defence-related class IV chitinases Planta 2004 219 4 626 638 15107993 10.1007/s00425-004-1268-8

[B14] de Lorenzo L Merchan F Blanchet S Megias M Frugier F Crespi M Sousa C Differential expression of the TFIIIA regulatory pathway in response to salt stress between *Medicago truncatula* genotypes Plant Physiol 2007 145 4 1521 1532 10.1104/pp.107.106146 17951460 PMC2151693

[B15] Merchan F Breda C Hormaeche JP Sousa C Kondorosi A Aguilar OM Megias M Crespi M A Kruppel-like transcription factor gene is involved in salt stress responses in Medicago spp Plant Soil 2003 257 1 1 9

[B16] Li G Wang BL Tian QY Wang TZ Zhang WH *Medicago truncatula* ecotypes A17 and R108 differed in their response to iron deficiency J Plant Physiol 2014 171 639 647 10.1016/j.jplph.2013.12.018 24709157

[B17] McNally KL Childs KL Bohnert R Davidson RM Zhao K Ulat VJ Zeller G Clark RM Hoen DR Bureau TE Stokowski R Ballinger DG Frazer KA Cox DR Padhukasahasram B Bustamante CD Weigel D Mackill DJ Bruskiewich RM Ratsch G Buell CR Leung H Leach JE Genomewide SNP variation reveals relationships among landraces and modern varieties of rice Proc Natl Acad Sci U S A 2009 106 30 12273 12278 10.1073/pnas.0900992106 19597147 PMC2718348

[B18] Clark RM Schweikert G Toomajian C Ossowski S Zeller G Shinn P Warthmann N Hu TT Fu G Hinds DA Chen HM Frazer KA Huson DH Schoelkopf B Nordborg M Raetsch G Ecker JR Weigel D Common sequence polymorphisms shaping genetic diversity in Arabidopsis thaliana Science 2007 317 5836 338 342 10.1126/science.1138632 17641193

[B19] Lai JS Li RQ Xu X Jin WW Xu ML Zhao HN Xiang ZK Song WB Ying K Zhang M Jiao YP Ni PX Zhang JG Li D Guo XS Ye KX Jian M Wang B Zheng HS Liang HQ Zhang XQ Wang SC Chen SJ Li JS Fu Y Springer NM Yang HM Wang JA Dai JR Schnable PS Genome-wide patterns of genetic variation among elite maize inbred lines Nat Genet 2010 42 11 1027 U1158 10.1038/ng.684 20972441

[B20] Borevitz JO Liang D Plouffe D Chang HS Zhu T Weigel D Berry CC Winzeler E Chory J Large-scale identification of single-feature polymorphisms in complex genomes Genome Res 2003 13 3 513 523 10.1101/gr.541303 12618383 PMC430246

[B21] Bruce M Hess A Bai JF Mauleon R Diaz MG Sugiyama N Bordeos A Wang GL Leung H Leach JE Detection of genomic deletions in rice using oligonucleotide microarrays BMC Genomics 2009 10 129 10.1186/1471-2164-10-129 19320995 PMC2666768

[B22] Amtmann A Learning from evolution: Thellungiella generates new knowledge on essential and critical components of abiotic stress tolerance in plants Mol Plant 2009 2 1 3 12 10.1093/mp/ssn094 19529830 PMC2639741

[B23] Wong CE Li Y Labbe A Guevara D Nuin P Whitty B Diaz C Golding GB Gray GR Weretilnyk EA Griffith M Moffatt BA Transcriptional profiling implicates novel interactions between abiotic stress and hormonal responses in Thellungiella, a close relative of Arabidopsis Plant Physiol 2006 140 4 1437 1450 10.1104/pp.105.070508 16500996 PMC1435811

[B24] Gong QQ Li PH Ma SS Rupassara SI Bohnert HJ Salinity stress adaptation competence in the extremophile *Thellungiella halophila* in comparison with its relative *Arabidopsis thaliana* Plant J 2005 44 5 826 839 10.1111/j.1365-313X.2005.02587.x 16297073

[B25] Wu HJ Zhang ZH Wang JY Oh DH Dassanayake M Liu BH Huang QF Sun HX Xia R Wu YR Wang YN Yang Z Liu Y Zhang WK Zhang HW Chu JF Yan CY Fang S Zhang JS Wang YQ Zhang FX Wang GD Lee SY Cheeseman JM Yang BC Li B Min JM Yang LF Wang J Chu CC Insights into salt tolerance from the genome of Thellungiella salsuginea Proc Natl Acad Sci U S A 2012 109 30 12219 12224 10.1073/pnas.1209954109 22778405 PMC3409768

[B26] Ryan PR Delhaize E Jones DL Function and mechanism of organic anion exudation from plant roots Annu Rev Plant Phys 2001 52 527 560 10.1146/annurev.arplant.52.1.527 11337408

[B27] Ma JF Role of organic acids in detoxification of aluminum in higher plants Plant Cell Physiol 2000 41 4 383 390 10.1093/pcp/41.4.383 10845450

[B28] Tester M Davenport R Na^+^ tolerance and Na^+^ transport in higher plants Ann Bot-London 2003 91 5 503 527 10.1093/aob/mcg058 PMC4242248 12646496

[B29] Blondon F Marie D Brown S Kondorosi A Genome size and base composition in *Medicago sativa* and *M. truncatula* species Genome 1994 37 2 264 270 10.1139/g94-037 18470076

[B30] Lam HM Xu X Liu X Chen WB Yang GH Wong FL Li MW He WM Qin N Wang B Li J Jian M Wang JA Shao GH Wang J Sun SSM Zhang GY Resequencing of 31 wild and cultivated soybean genomes identifies patterns of genetic diversity and selection Nat Genet 2010 42 12 1053 U1041 10.1038/ng.715 21076406

[B31] Furukawa J Yamaji N Wang H Mitani N Murata Y Sato K Katsuhara M Takeda K Ma JF An aluminum-activated citrate transporter in barley Plant Cell Physiol 2007 48 8 1081 1091 10.1093/pcp/pcm091 17634181

[B32] Magalhaes JV Liu J Guimaraes CT Lana UGP Alves VMC Wang YH Schaffert RE Hoekenga OA Pineros MA Shaff JE Klein PE Carneiro NP Coelho CM Trick HN Kochian LV A gene in the multidrug and toxic compound extrusion (MATE) family confers aluminum tolerance in sorghum Nat Genet 2007 39 9 1156 1161 10.1038/ng2074 17721535

[B33] Liu JP Magalhaes JV Shaff J Kochian LV Aluminum-activated citrate and malate transporters from the MATE and ALMT families function independently to confer Arabidopsis aluminum tolerance Plant J 2009 57 3 389 399 10.1111/j.1365-313X.2008.03696.x 18826429

[B34] Maron LG Pineros MA Guimaraes CT Magalhaes JV Pleiman JK Mao CZ Shaff J Belicuas SNJ Kochian LV Two functionally distinct members of the MATE (multi-drug and toxic compound extrusion) family of transporters potentially underlie two major aluminum tolerance QTLs in maize Plant J 2010 61 5 728 740 10.1111/j.1365-313X.2009.04103.x 20003133

[B35] Curie C Cassin G Couch D Divol F Higuchi K Jean M Misson J Schikora A Czernic P Mari S Metal movement within the plant: contribution of nicotianamine and yellow stripe 1-like transporters Ann Bot-London 2009 103 1 1 11 10.1093/aob/mcn207 PMC2707284 18977764

[B36] Waters BM Chu HH DiDonato RJ Roberts LA Eisley RB Lahner B Salt DE Walker EL Mutations in Arabidopsis *Yellow Stripe-Like1* and *Yellow Stripe-Like3* reveal their roles in metal ion homeostasis and loading of metal ions in seeds Plant Physiol 2006 141 4 1446 1458 10.1104/pp.106.082586 16815956 PMC1533956

[B37] Fowler S Thomashow MF Arabidopsis transcriptome profiling indicates that multiple regulatory pathways are activated during cold acclimation in addition to the CBF cold response pathway Plant Cell 2002 14 8 1675 1690 10.1105/tpc.003483 12172015 PMC151458

[B38] Hao QN Zhou XA Sha AH Wang C Zhou R Chen SL Identification of genes associated with nitrogen-use efficiency by genome-wide transcriptional analysis of two soybean genotypes BMC Genomics 2011 12 525 10.1186/1471-2164-12-525 22029603 PMC3210170

[B39] Alba R Fei ZJ Payton P Liu Y Moore SL Debbie P Cohn J D’Ascenzo M Gordon JS Rose JKC Martin G Tanksley SD Bouzayen M Jahn MM Giovannoni J ESTs, cDNA microarrays, and gene expression profiling: tools for dissecting plant physiology and development Plant J 2004 39 5 697 714 10.1111/j.1365-313X.2004.02178.x 15315633

[B40] Kang Y Han YH Torres-Jerez I Wang MY Tang YH Monteros M Udvardi M System responses to long-term drought and re-watering of two contrasting alfalfa varieties Plant J 2011 68 5 871 889 10.1111/j.1365-313X.2011.04738.x 21838776

[B41] Nordborg M Weigel D Next-generation genetics in plants Nature 2008 456 7223 720 723 10.1038/nature07629 19079047

[B42] Yoder JB Briskine R Mudge J Farmer A Paape T Steele K Weiblen GD Bharti AK Zhou P May GD Young ND Tiffin P Phylogenetic Signal Variation in the Genomes of Medicago (Fabaceae) Syst Biol 2013 62 3 424 438 10.1093/sysbio/syt009 23417680

[B43] Kochian LV Cellular mechanisms of aluminum toxicity and resistance in plants Annu Rev Plant Phys 1995 46 237 260 10.1146/annurev.pp.46.060195.001321

[B44] Deyholos MK Sieburth LE Separable whorl-specific expression and negative regulation by enhancer elements within the *AGAMOUS* second intron Plant Cell 2000 12 10 1799 1810 10.1105/tpc.12.10.1799 11041877 PMC149120

[B45] Gidekel M Jimenez B HerreraEstrella L The first intron of the *Arabidopsis thaliana* gene coding for elongation factor 1 beta contains an enhancer-like element Gene 1996 170 2 201 206 10.1016/0378-1119(95)00837-3 8666245

[B46] Merchan F de Lorenzo L Rizzo SG Niebel A Manyani H Frugier F Sousa C Crespi M Identification of regulatory pathways involved in the reacquisition of root growth after salt stress in *Medicago truncatula* Plant J 2007 51 1 1 17 10.1111/j.1365-313X.2007.03117.x 17488237

[B47] Urao T Yamaguchishinozaki K Urao S Shinozaki K An Arabidopsis *myb* homolog is induced by dehydration stress and its gene-product binds to the conserved MYB recognition sequence Plant Cell 1993 5 11 1529 1539 10.1105/tpc.5.11.1529 8312738 PMC160383

[B48] Nakashima K Fujita Y Katsura K Maruyama K Narusaka Y Seki M Shinozaki K Yamaguchi-Shinozaki K Transcriptional regulation of ABI3-and ABA-responsive genes including *RD29B* and *RD29A* in seeds, germinating embryos, and seedlings of Arabidopsis Plant Mol Biol 2006 60 1 51 68 10.1007/s11103-005-2418-5 16463099

[B49] Simpson SD Nakashima K Narusaka Y Seki M Shinozaki K Yamaguchi-Shinozaki K Two different novel cis-acting elements of erd1, a *clpA* homologous Arabidopsis gene function in induction by dehydration stress and dark-induced senescence Plant J 2003 33 2 259 270 10.1046/j.1365-313X.2003.01624.x 12535340

[B50] Chinnusamy V Ohta M Kanrar S Lee BH Hong XH Agarwal M Zhu JK ICE1: a regulator of cold-induced transcriptome and freezing tolerance in Arabidopsis Gene Dev 2003 17 8 1043 1054 10.1101/gad.1077503 12672693 PMC196034

[B51] Chen WQ Provart NJ Glazebrook J Katagiri F Chang HS Eulgem T Mauch F Luan S Zou GZ Whitham SA Budworth PR Tao Y Xie ZY Chen X Lam S Kreps JA Harper JF Si-Ammour A Mauch-Mani B Heinlein M Kobayashi K Hohn T Dangl JL Wang X Zhu T Expression profile matrix of Arabidopsis transcription factor genes suggests their putative functions in response to environmental stresses Plant Cell 2002 14 3 559 574 10.1105/tpc.010410 11910004 PMC150579

[B52] Curie C Panaviene Z Loulergue C Dellaporta SL Briat JF Walker EL Maize yellow stripe1 encodes a membrane protein directly involved in Fe(III) uptake Nature 2001 409 6818 346 349 10.1038/35053080 11201743

[B53] Vonwiren N Mori S Marschner H Romheld V Iron inefficiency in maize mutant *ys1* (*Zea mays* L. Cv Yellow-Stripe) is caused by a defect in uptake of iron phytosiderophores Plant Physiol 1994 106 1 71 77 12232304 10.1104/pp.106.1.71 PMC159500

[B54] DiDonato RJ Roberts LA Sanderson T Eisley RB Walker EL Arabidopsis *Yellow Stripe-Like2 (YSL2)*: a metal-regulated gene encoding a plasma membrane transporter of nicotianamine-metal complexes Plant J 2004 39 3 403 414 10.1111/j.1365-313X.2004.02128.x 15255869

[B55] Conte SS Walker EL Transporters contributing to iron trafficking in plants Mol Plant 2011 4 3 464 476 10.1093/mp/ssr015 21447758

[B56] Wang Z Shen J Zhang F Cluster-root formation, carboxylate exudation and proton release of *Lupinus pilosus* Murr. as affected by medium pH and P deficiency Plant Soil 2006 287 1–2 247 256

[B57] Ewing B Green P Base-calling of automated sequencer traces using phred. II. Error probabilities Genome Res 1998 8 3 186 194 9521922

[B58] Ewing B Hillier L Wendl MC Green P Base-calling of automated sequencer traces using phred. I. Accuracy assessment Genome Res 1998 8 3 175 185 10.1101/gr.8.3.175 9521921

[B59] Li R Yu C Li Y Lam TW Yiu SM Kristiansen K Wang J SOAP2: an improved ultrafast tool for short read alignment Bioinformatics 2009 25 15 1966 1967 10.1093/bioinformatics/btp336 19497933

[B60] Higo K Ugawa Y Iwamoto M Korenaga T Plant cis-acting regulatory DNA elements (PLACE) database: 1999 Nucleic Acids Res 1999 27 1 297 300 10.1093/nar/27.1.297 9847208 PMC148163

