# Supplementary figures and images for: Genome variations account for different response to three mineral elements between Medicago truncatula ecotypes Jemalong A17 and R108
Source: BMC Plant Biol. 2014 May 6;14:122. doi: 10.1186/1471-2229-14-122 (PMC4031900; doi:10.1186/1471-2229-14-122)

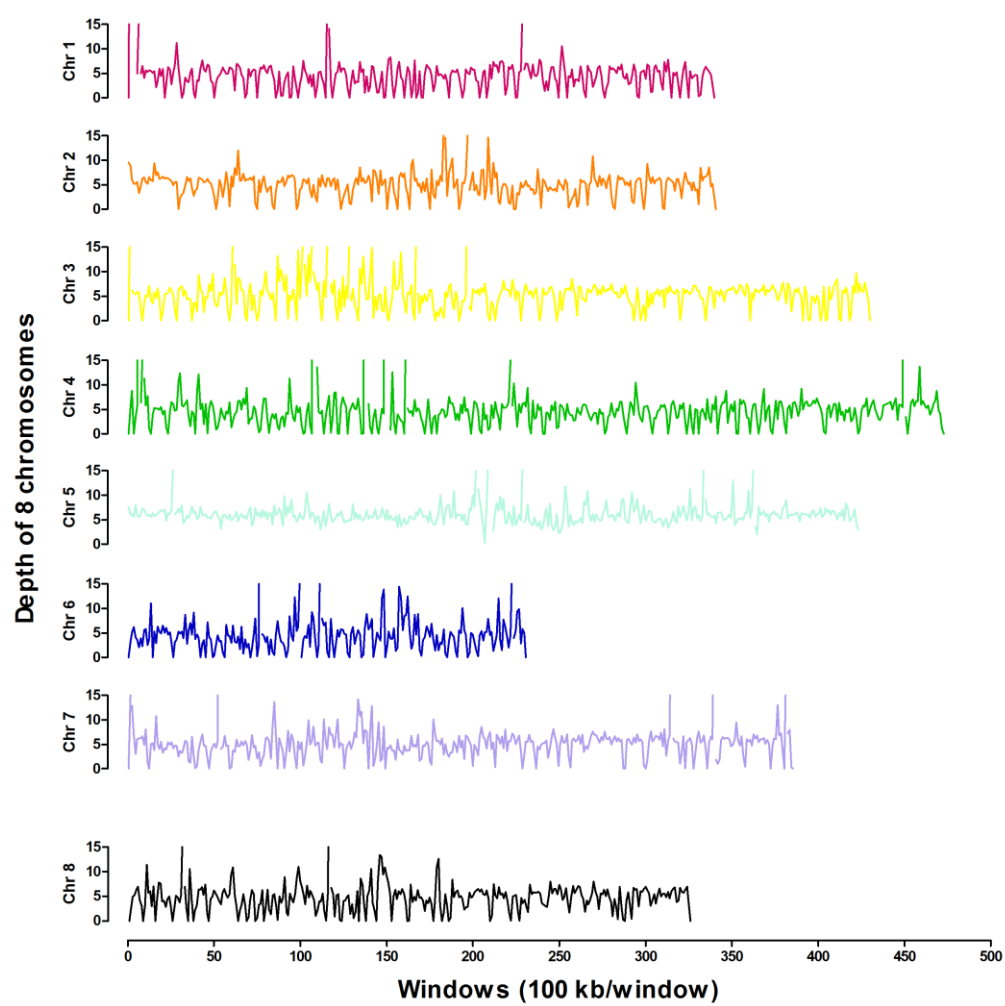

**Additional figure 1**

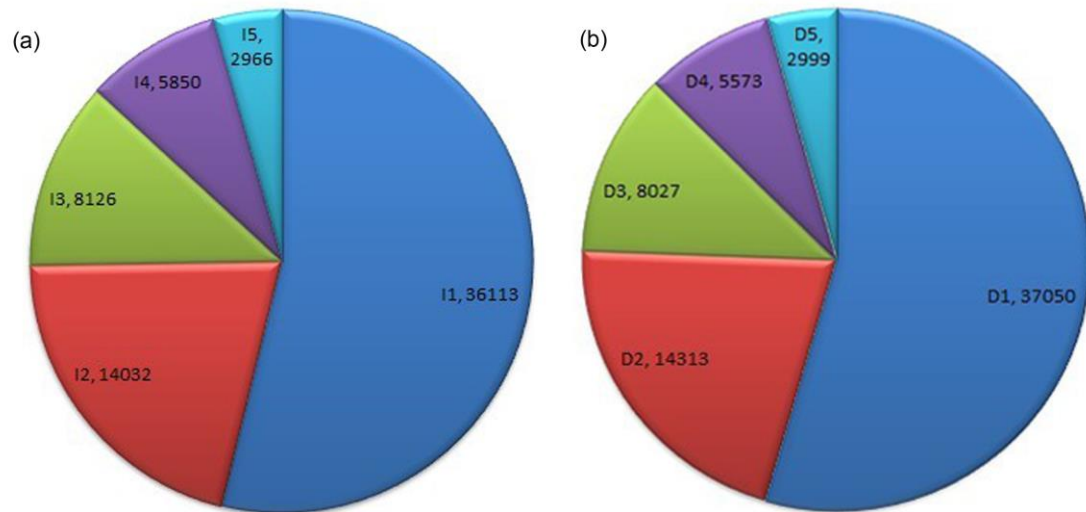

**Additional figure 2**

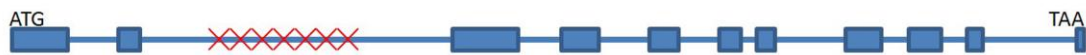

**Additional figure 3**

Supplement: Additional file 1: Figure S1 — The R108 sequencing depth of 8 chromosomes against to the reference J. A17. One hundred kb was defined as one window. The points with depth more than 15 were hided to make the figure clearer. Figure S2. The number of indels varying from 1 to 5 bp in the genome of R108. The number of insertions and deletions varying from 1 to 5 bp was shown in panel (a) and (b), respectively. The “I” and “D” mean insertion and deletion, respectively. Figure S3. The structure of the MtAACT genomic region. The exons and introns are drawn as rectangles and lines, respectively. The region with red crosses is deleted in the genome of R108. [file 1471-2229-14-122-S1.pdf]
